# Light-Induced Control of the Spin Distribution on Cu–Dithiolene Complexes: A Correlated Ab Initio Study

**DOI:** 10.3390/molecules24061088

**Published:** 2019-03-19

**Authors:** Jhon Zapata-Rivera, Carmen J. Calzado

**Affiliations:** 1 Facultad de Ciencias Básicas and Universidad Tecnológica de Bolívar, Campus Tecnológico s/n, 131001 Cartagena, Colombia; jzapatarivera@icloud.com; 2 Departamento de Química Física, Universidad de Sevilla, c/Prof. García González and s/n, 41012 Sevilla, Spain

**Keywords:** spin control, magnetism, dmit radicals, UV–Vis spectrum, CASSCF/CASPT2 calculations

## Abstract

Metal dithiolene complexes—M(dmit)_2_—are key building blocks for magnetic, conducting, and optical molecular materials, with singular electronic structures resulting from the mixing of the metal and dmit ligand orbitals. Their use in the design of magnetic and conducting materials is linked to the control of the unpaired electrons and their localized/delocalized nature. It has been recently found that UV–Vis light can control the spin distribution of some [Cu(dmit)_2_]^−2^ salts in a direct and reversible way. In this work, we study the optical response of these salts and the origin of the differences observed in the EPR spectra under UV–Vis irradiation by means of wave function-based quantum chemistry methods. The low-lying states of the complex have been characterized and the electronic transitions with a non-negligible oscillator strength have been identified. The population of the corresponding excited states promoted by the UV–Vis absorption produces significant changes in the spin distribution, and could explain the changes observed in the system upon illumination. The interaction between neighbor [Cu(dmit)_2_]^−2^ complexes is weakly ferromagnetic, consistent with the relative orientation of the magnetic orbitals and the crystal packing, but in disagreement with previous assignments. Our results put in evidence the complex electronic structure of the [Cu(dmit)_2_]^−2^ radical and the relevance of a multideterminantal approach for an adequate analysis of their properties.

## 1. Introduction

The search for molecular systems showing simultaneously conduction and magnetism has attracted the attention of many research groups for a long time [1,2,3]. Both properties require the presence of unpaired electrons in the system, but of different nature: localized in the case of magnetism, while conduction is associated to itinerant electrons. To realize such molecular magnetic conductors, a common strategy consists in using hybrid compounds mixing the carriers of the organic part with the magnetic centers of transition metal complexes. However, in most cases, the resulting material is a nonmagnetic insulator, since the magnetic centers are isolated by the bulky organic molecules, and show negligible interactions between carriers. In fact, the number of reported molecular magnetic conductors is rather limited [4,5,6,7,8,9,10].

An alternative consists in generating in situ the carriers and/or spins using light irradiation. This *optical doping* is the procedure employed in systems based on metal–dithiolene M(dmit)_2_ complexes. In fact, metal–dithiolene complexes have increasingly developed over the last years due to their singular electronic structures, related to both the redox activity of the dmit ligand and the metal center and the dmit–metal hybridization. These features make these complexes notable building blocks for magnetic, conducting, and optical molecular materials. They exhibit exceptional physical properties including ferromagnetism [11], conductivity [3,12,13], superconductivity [14], chirality [15], electrocatalytic, and photocatalytic water splitting ability [16,17], and represent ideal units to reach multifunctionality [3,12,15,18,19]. Systems such as MV[Ni(dmit)_2_]_2_ and BPY[Ni(dmit)_2_]_2_ (dmit = 1,3-dithiole-2-thione-4,5-dithiolate, BPY^+2^ = 2,2′-bipyridine dication, MV^+2^ = methylviologen dication) are called photomagnetic conductors [20,21]. They are nonmagnetic insulators in the dark, and become conductors with magnetic behavior under UV irradiation. The photocarriers are produced by charge transfer (CT) excitations between the Ni complex and the organic cations occurring in the UV region. This introduces unpaired electrons in the organic cations and significantly enhances the conductivity of the salts. 

Recently, a similar optical doping has been reported for a family of three hybrid salts based on the [Cu(dmit)_2_]^−2^ complex, synthesized, and characterized by Noma et al. [22,23,24]. In these cases, there is not a net charge transfer between cations and anions, but the UV irradiation produces reversible changes in the spin distributions of the anions that manifest in the signals of the EPR spectra, different in the dark and under UV-irradiated conditions. Once the UV irradiation ceases, the EPR spectra recover their original shape, confirming the reversibility of the process. There then exists a direct and reversible optical control of the spin distribution [22,23,24].

It is the aim of this work to study in detail the optical response of these hybrid salts based on [Cu(dmit)_2_]^−2^ and the origin of the differences observed in the EPR spectra under UV irradiation by means of wave function-based quantum chemistry methods. CASSCF and CASPT2 calculations on the basis of extended active spaces have been performed to analyze the electronic structure of the ground state of the [Cu(dmit)_2_]^−2^ complex and the nature and energy of the excited states, that can be optically populated with light in the UV–Vis range. Our results show the presence of a set of excited states accessible by UV–Vis irradiation, with different nature than the ground state, which can explain the differences observed in the EPR spectra under irradiation. This study provides an alternative interpretation of the reported experimental data of Noma et al. [22,23,24] and insights on the physics effects governing the photocontrol of the spin distribution in this compound. The relevance of this study is not just constrained to this particular system, but can also help in interpreting the properties of related metal–dithiolene compounds. 

## 2. Results

Naito and coworkers [23,24] reported on three salts of [Cu(dmit)_2_]^−2^ with reversible optical control of the spin distribution. They are [n-Bu_4_N]_2_[Cu(dmit)_2_], [(DABCO)H]_2_[Cu(dmit)_2_]CH_3_CN, and BP_2_DBF[Cu(dmit)_2_] (**1**, **2**, and **3,** respectively, in the works by Naito et al. [23,24]), with n-Bu_4_N = tetrabutylammonium, BP_2_DBF^2+^ = dibenzofuran-2,2′-bis(*N*-methylene-4,4′-bipyridinium), and DABCO = 1,4-diazabicyclo[2 .2.2]octane). They differ on the counter cation and slightly on the molecular structure of the [Cu(dmit)_2_]^−2^ anion, square planar in **1**, slightly distorted square planar for **2**, and a distorted tetrahedral coordination for **3**. All possess S = 1/2 on the [Cu(dmit)_2_]^−2^ units, and exhibit similar UV–Vis spectra and electron paramagnetic resonance (EPR) spectra under dark and UV–Vis irradiated conditions. Then the photoinduced changes are neither related to the specific cation nor to the packing features of the salt, although in the case of **3** the UV–Vis illumination promotes charge transfer transitions from the anion to the cation and the EPR spectra is a bit more complicate due to the presence of unpaired electrons also in the cations. Our study focuses on the tetrabutylammonium salt [n-Bu_4_N]_2_[Cu(dmit)_2_], but the conclusions are then relevant for the two other compounds.

**Figure 1:**
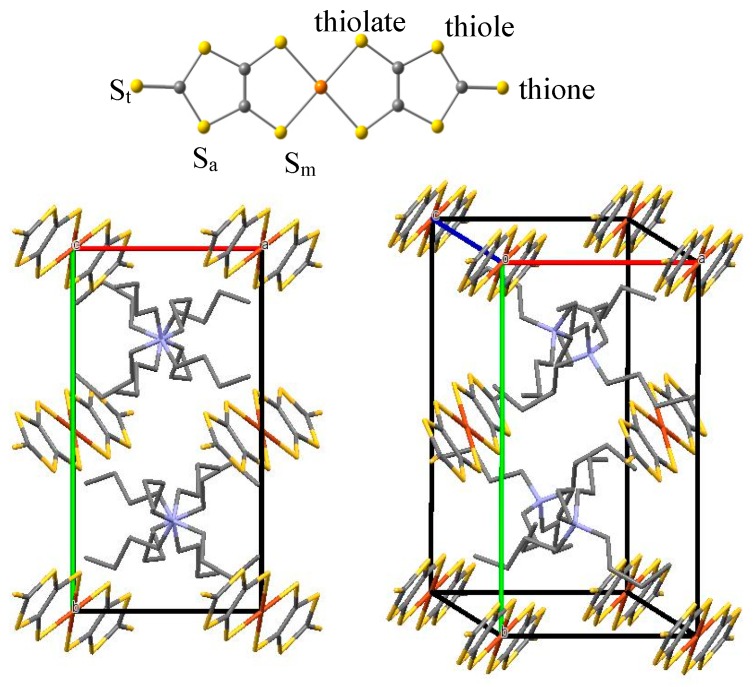
(**top**) Molecular structure of the planar [Cu(dmit)_2_]^−2^ complex, with the notation employed for the S atoms. (**bottom**) View of the [n-Bu_4_N]_2_[Cu(dmit)_2_] crystal along *b* axis (**left**) and 3D view of the crystal (**right**), where the H atoms have been omitted. Yellow, orange, gray, and blue wires (balls) correspond to S, Cu, C, and N atoms, respectively.

This salt crystallizes in the monoclinic space group P2_1_/c, with two molecules per unit cell [23] (Figure 1). The n-Bu_4_N^+^ and [Cu(dmit)_2_]^−2^ species form alternating layers along the crystallographic *b* axis. The [Cu(dmit)_2_]^−2^ anions adopt a parallel arrangement along the *a* axis, separated by a large Cu…Cu distance of 8.466 Å. The shortest intermolecular S…S contact is as large as 6.14 Å, larger than twice the van der Waals S radius (2 × 1.85 Å = 3.70 Å). This suggests a very weak orbital interaction between the adjacent anions, and consequently that the material should be an insulator [23]. There are two equivalent anionic layers, alternating with the cationic layers, where the [Cu(dmit)_2_] species in different layer are orthogonal. The complex anion is nearly planar, the Cu atom occupying an inversion center (local C_i_ point group). The average Cu–S bond distance inside each [Cu(dmit)_2_]^−2^ complex is of 2.29 Å, in agreement with those found in other [M(L)_2_]^n-^ complexes, with M = Cu, Ni, Pd, Pt, and L being dithiolate-type ligands [24,25].

**Figure 2:**
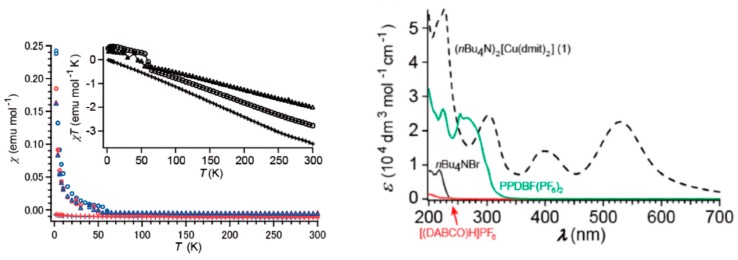
(**left**) Temperature dependence of the magnetic susceptibility of [n-Bu_4_N]_2_[Cu(dmit)_2_] (circles) and two related compounds: [(DABCO)H]_2_[Cu(dmit)_2_]CH_3_CN (crosses) and BP_2_DBF[Cu(dmit)_2_] (triangles). Blue and red circles correspond to field-cooling and zero-field-cooling measurements, respectively. (**right**) UV–Vis absorption spectra of [n-Bu_4_N]_2_[Cu(dmit)_2_] in CH_3_CN at 20 °C. The UV–Vis-near infrared (NIR) diffuse reflectance spectra in KBr differ by the relative intensity of the bands, but not for the position of these bands. From Ref. [23].

In the [Cu(dmit)_2_]^−2^ complexes, the ligands are formally closed-shell dmit^−2^ and the metal atoms are Cu(II), with a d^9^ configuration. Hence each [Cu(dmit)_2_]^−2^ complex has one unpaired electron. The thermal dependence of the magnetic susceptibility measured on polycrystalline samples [23] presented a Curie-like behavior up to 50 K, and a diamagnetic contribution for T >50 K of −1.0 10^−2^ emu mol^−1^ (Figure 2). The paramagnetic part of the curve (T < 50 K) has been attributed to the presence of magnetic impurities, such as oxygen adsorbed on the sample, and the diamagnetic contribution was related to the strongly antiferromagnetic interaction between the [Cu(dmit)_2_]^−2^ complexes [23,24], despite the long intermolecular distances (Cu…Cu 8.466 Å, shortest S…S contact of 6.138 Å). The EPR spectrum under dark conditions was symmetric without fine structure and average g-values of 2.02 to 2.04. The fact that the hyperfine structure was not observed was related to the broadening effect of the intermolecular antiferromagnetic interactions and spin-orbit coupling on the sulfur atoms of the ligands [23,24]. The UV–Vis absorption spectra of [n-Bu_4_N]_2_[Cu(dmit)_2_] in CH_3_CN presented bands at 530, 398, 302, and 226 nm, almost equivalent to those obtained by UV–Vis-near infrared (NIR) diffuse reflectance spectra in KBr pellets (Figure 2 and Refs. [23,24]). Under UV–Vis irradiation, the EPR signals showed a marked difference with respect to those in the dark: higher intensities, hyperfine structure, and signals shifted to higher resonance magnetic fields. The observed g-values under UV irradiation (g = ~2.00) were smaller than those under dark conditions. These features were interpreted as due to the increase of the Cu contribution to the spin density promoted by the UV irradiation [22,23], although these g-values are smaller than those generally observed for Cu(II) complexes [26,27,28,29,30,31]. The system is an insulator in the dark and did not exhibit any response under UV irradiation.

This set of results clearly indicates the optically-induced modification of the sample, but the reported interpretation [22,23,24] does not seem to be satisfactory, even contradictory in some points. Wave function based quantum chemistry approaches were used to analyze the electronic structure of the [Cu(dmit)_2_]^−2^ complex in the ground state and in those excited states accessible by UV–Vis irradiation. Additionally, the magnetic coupling interaction between [Cu(dmit)_2_]^−2^ complexes was also evaluated. The aim of this study is three-fold: (i) to determine the nature of the SOMO in the ground state and the spin distribution responsible for the EPR signals in the dark conditions; (ii) to simulate the UV–Vis absorption spectra and figure out the excitations involving a change on the spin distribution of the [Cu(dmit)_2_]^−2^, related to the changes observed in EPR under UV irradiation; and (iii) to evaluate the amplitude of the interactions between unpaired electrons and offer an alternative interpretation of the χ vs. T curve.

### 2.1. Ground State Wave Function of the Cu(dmit)_2_ Complex

Dithiolene ligand is a well-known noninnocent ligand able to present different oxidation states depending on the metal to which it is bound [3,32,33,34]. This is related to the bonding scheme between the metal and dithiolene ligands in the [M(dithiolene)_2_]^−n^ complexes, which can be a normal bonding scheme, with the metal orbitals destabilized with respect to the ligand orbitals, or the opposite, called the inverted bonding scheme. In the former case, the highest-occupied molecular orbitals (HOMO) or the single occupied molecular orbitals (SOMO) if the complex is a radical has a dominant metal contribution, while in the case of the inverted bonding scheme, the SOMO is mainly composed by the ligand orbitals [13,35,36]. Different experimental techniques such as XAS, ENDOR/ESEEM, and EPR spectroscopies [35,37,38], as well as quantum chemistry calculations (DFT, CASSCF/PT2) [36,39] on [Ni(dithiolene)_2_]^−n^ and [Cu(dithiolene)_2_]^−n^ complexes present evidences of an inverted bonding scheme in most of the Ni complexes, while Cu ones usually have a normal bonding.

**Figure 3:**
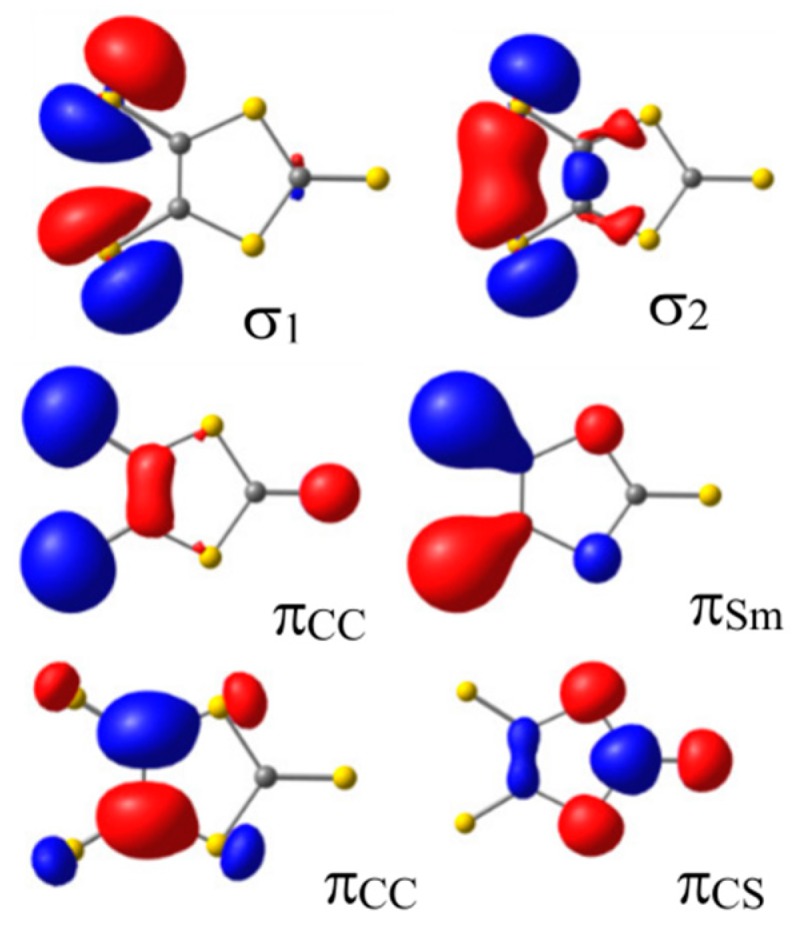
Valence occupied and virtual σ and π orbitals of dmit^−2^ ligand that can be combined with M 3d orbitals.

Three type of S atoms can be distinguished in the dmit ligand (Figure 1): S_m_ is a thiolate sulfur, S_a_ a thiole sulfur, and S_t_ a thione sulfur [40]. The valence orbitals of the dmit^−2^ ligand are largely localized on the π and σ lone pair S orbitals. Figure 3 shows six of these orbitals, occupied (σ_1_, σ_2_, π_CC_, and π_Sm_) and empty (π_CS_* and π_CC_*) in the ground state. The π_CS_* orbital is mainly a combination of the antibonding C = S_t_ orbital with the 3pz orbitals of the thiole sulfur S_a_ atoms. Similarly, the π_CC_* is mainly the antibonding combination of the 2pz orbitals on atoms C1–C2. In the M(dmit)_2_ complex, these MOs can mix in different extent with the M 3d orbitals, depending on the metal, the formal charge of the complex, the crystal structure, and the packing constraints imposed by the supramolecular cations.

In the case of the [*n*-Bu_4_N]_2_[Cu(dmit)_2_], the cation has a closed-shell configuration and the unpaired electron is completely localized on the anion. It is well known that the singly occupied molecular orbital SOMO of [Cu(dmit)_2_]^−2^ anions have much higher degree of π-d mixing than that in other metal–dithiolene complex anions [27,36,38,41]. Thus, it has been reported a Cu 3d_xy_: ligand ratio of 36:64 for the SOMO of Cu–Dithiolene complexes with distorted tetrahedral coordination [27]. In our case, the nature of the SOMO of the [Cu(dmit)_2_]^−2^ complex has been determined by CASSCF calculations on the doublet Ag ground state. Using the simplest possible CAS, one electron in one MO, i.e., a restricted open-shell calculation, the SOMO results to be the antibonding combination of the Cu 3d_xy_ orbital and the σ_1_−σ_1_ dmit orbital, with a 70:30 mixing of Cu and dmit, respectively. The description of the ground state is qualitatively the same, regardless of the size and composition of the CAS. In fact, increasing progressively the size of the CAS, including additional 3d Cu and σ and π dmit orbitals do not modify in a significant manner the description of the ground state.

**Figure 4:**
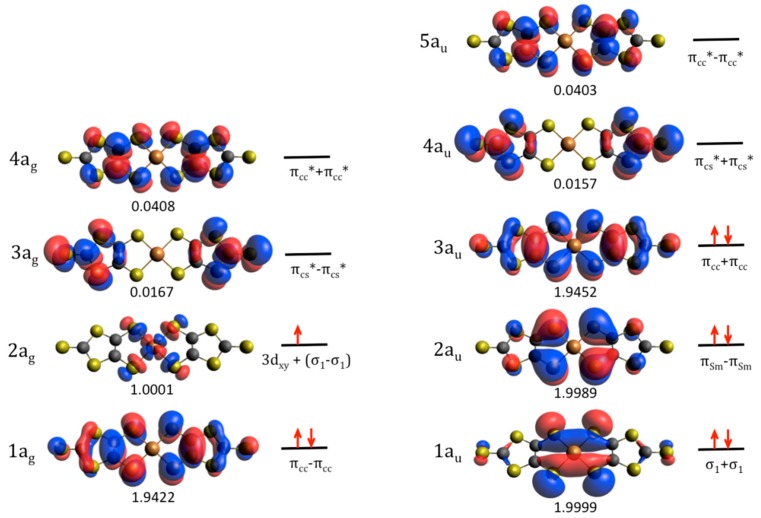
Active orbitals employed in the CASSCF/CASPT2 calculations of the ground and excited doublet states of the [Cu(dmit)_2_]^−2^ complex and occupation number of the natural active orbitals for the ground state. The red arrows represent the occupation of the active MOs on the dominant electronic configuration (84% of the weight) of the ^2^Ag ground state.

Many test calculations have been performed with active spaces of different composition and number of electrons (see Tables S3 and S4 and Figure S1). The d → d excitations are out of the range of the explored UV–Vis range (Table S3) and in addition they are electric-dipole-forbidden transitions. Then these excitations are not (significantly) contributing to the recorded UV–Vis spectra of these salts. Once we have confirmed that the presence of 3d orbitals on the active space does not modify significantly the description of the ground state, the criterion for choosing the CAS is to introduce significant orbitals for describing the low-lying excited states, but retaining the same composition for both sets of Ag and Au states. Figure 4 contains the occupation and composition of the active natural orbitals resulting from a CASSCF(9/9) calculation on the ^2^Ag ground state. This active space, with nine electrons in nine MOs, represents the best compromise between accuracy and feasibility, and includes all the orbitals playing a role on the description of the excited states accessible by electronic excitations in the UV–Vis range.

**Figure 5:**
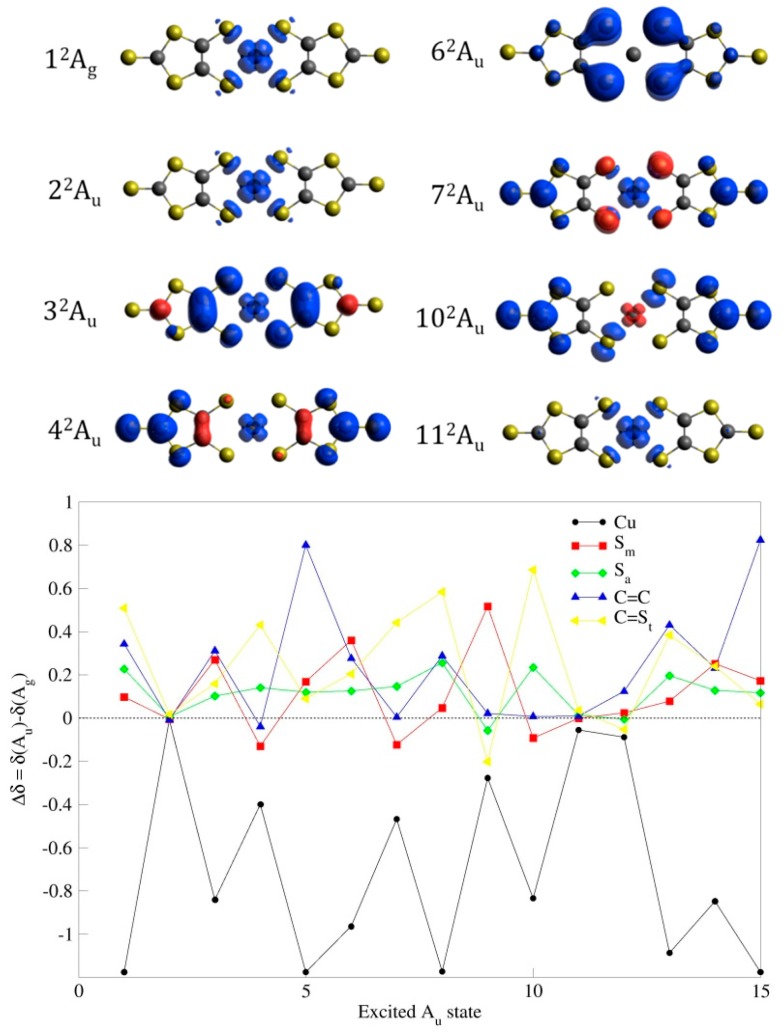
(**top**) Spin density maps for the ground state and excited A_u_ states involved in the main UV–Vis absorption bands. (**bottom**) Changes in the spin populations on Cu atom (black), S_m_ atoms (red), S_a_ atoms (green), C1–C2 atoms (blue), and C=S_t_ atoms (yellow) between the ground state and the different Au excited states.

The ^2^Ag ground state is mainly described by the (π_Sm_ − π_Sm_)^2^(σ_Sm_ + σ_Sm_)^2^(π_cc_ + π_cc_)^2^(π_CC_ − π_CC_)^2^(SOMO) configuration (84% of the weight, Figure 4). The SOMO, as in the restricted open-shell Hartee–Fock (ROHF) description, is a 65:35 mixing of the 3d_xy_ and σ_1_-σ_1_ orbitals, respectively, and with the same shape that the SOMO provided by Noma et al. [23] from extended Hückel calculations. The virtual (π_CS_* + π_CS_*) and (π_CC_* − π_CC_*) MOs belong to the a_u_ representation, while (π_CS_* − π_CS_*) and (π_CC_* + π_CC_*) belong to the a_g_ one. For the ground state, the spin density on the Cu atom is 0.891, mostly located on the 3d_xy_ orbital, as shown in Figure 5.

The nature of the SOMO resulting from the CASSCF calculations is in line with the picture provided by the ^33^S super-hyperfine interactions observed in the single crystal EPR spectra of the salt [38]. They suggested large mixing between the Cu and S atoms (almost 50:50) in the molecular orbital carrying this unpaired electron. This high degree of covalency of the Cu–S bonds is also found for other Cu(II) complexes having a planar CuS_4_ coordination sphere [38]. In the case of the related [Cu(mnt)_2_]^−2^ complex, with mnt = maleonitriledithiolate, the intensity of the Cu L-edge bands in the X-ray absorption spectroscopy (XAS) spectra suggested a total Cu character of 39 ± 3% in the SOMO orbital [37] that goes up to 45% when using the ^33^S super-hyperfine EPR data. DFT calculations on the optimized geometry of the [Cu(mnt)_2_]^−2^ result in a 38–45% of Cu character of the SOMO employing the BP86 and B3LYP functionals, respectively.

It is important to mention the well-known over-delocalization of the DFT-based descriptions of the electronic structure, while CASSCF calculations are prone to overlocalize on the metal the singly occupied orbitals [42,43,44,45,46,47]. This manifests itself in the underestimation of the Cu 3d contribution at DFT level, and the overestimation at CASSCF one. A correlated description of the wave function, as the Difference Dedicated Configuration Interaction (DDCI) one, should provide a better description of the ground state, in particular, the relative weight of both components. In fact, this trend can already be observed from the singly occupied natural orbital resulting from the DDCI(1/1) wave function, i.e., a DDCI calculation on the top of the ROHF wave function. The natural active orbital at this level contains 61:39 of Cu:dmit weights, to be compared to the 70:30 mixing of the corresponding ROHF wave function. This effect is expected to be enhanced when using larger active spaces. Then the natural orbitals of a correlated wave function reduces the overlocalization of the active electrons on the metal and describe better the delocalization of the active electrons on the ligands [42].

In summary, the contribution of the Cu orbitals to the SOMO depends on the experimental procedure and also the theoretical approach employed to determine the electronic structure of the ground state. However, in all cases it is clearly evidenced that this orbital results from the non-negligible mixing between the Cu 3d_xy_ orbitals and the σ S orbitals of the dmit ligands.

### 2.2. Excited States

The lowest excited states of Au symmetry have been determined from state-average CASSCF/CASPT2 calculations. Their relative energies with respect to the ground Ag state at MS-CASPT2 level and associated wavelength are collected in Table 1, as well as the oscillator strength resulting from the RASSI approach. The composition of the wave function of these excited states is only shown when the oscillator strength (*f*) is larger than 1.0 × 10^−4^, since our purpose is to identify the excited states involved in the changes observed after irradiation. Most of the wave functions are highly multiconfigurational, only the dominant configurations are shown in Table 1, some additional contributions can be found in Table S1. The Mulliken spin population analysis is reported in Table S2. Comparing with the analysis resulting from the tight-binding band calculation carried out by Naito and coworkers [22,23] on the same system; it is important to highlight the fact that our approach provides a complete description of the low-lying excited states, i.e., wave functions and accurately calculated transition energies, including both dynamical and nondynamical correlation effects as well as the oscillator strength for each transition.

It is important to notice that the absolute value of the oscillator strength is rather sensitive to the strategy employed for its evaluation, with different absolute values depending on whether the CASPT2 or MS-CASPT2 wave functions are selected for the ground state. In contrast in all explored cases, the *relative* values of the different excitations maintain the same trends. The values discussed hereafter have been obtained employing the CASPT2 description of the ground state and the MS-CASPT2 description for the excited Au states.

There are two states which transitions from the ground state present very high *f* values. They correspond to the 2^2^Au and 11^2^Au states, separated by 2.25 and 4.80 eV to the ground state, respectively. The wavelengths of the associated transitions are 551.2 and 258.6 nm, respectively, in good agreement with the absorption bands at 530 and 226 nm, observed both in solution and in powder [23,24] (Figure 2). In these states, the Cu atom carries most of the spin density as in the ground state. In fact, Figure 4 shows almost a null difference between the spin density of the ground state and the 2^2^Au and 11^2^Au states. Then these two excitations are not related to the changes observed in the system upon UV–Vis irradiation.

Together with these two intense transitions, five additional excitations can be distinguished with *f* values one order of magnitude smaller. They correspond to the transitions to the 3^2^Au, 4^2^Au, 6^2^Au, 7^2^Au, and 10^2^Au states, all of them with energies in the range of 3.30 to 4.70 eV, and associated wavelengths of 375, 358, 306, 305, and 262 nm, respectively. They can be identified with the bands observed in the range of 300 to 400 nm, of different relative intensity in solution and powder. In all cases, the wave function of the excited state shows a significant reduction of the spin density on Cu atom with respect to the ground state, as shown numerically in Table 1 and graphically displayed by the spin density plots in Figure 5. Although the wave functions of these states are highly multideterminantal in nature, it is possible to analyze in a qualitative manner some of them. The spin density value can be estimated in a rather naive way by considering the spin distribution on the different determinants of the dominant configuration (Table 1), and their weights in the wave function. Notice that in our active space only the 2a_g_ orbital is a metal-centered orbital, the rest are mainly dmit-centered orbitals. Following this approach, the net spin density on the 2a_g_ orbital results from the sum of the weights of the determinants with spin up on the 2a_g_ orbital, minus the weight of determinants where the 2a_g_ orbital bears a spin down.

In the case of the 3^2^Au state, the dominant configuration contains three singly occupied MOs (2a_g_, 3a_g_, and 3a_u_), and could be roughly described as resulting from an excitation from the 3a_u_ (π_CC_ + π_CC_) to 3a_g_ (π_CS_* + π_CS_*) orbital with respect to the ground state. However this simplified description can hardly explain the change on the Cu spin density. In fact, it is important to take into account the multideterminantal nature of this excitation. Notice that the 2a_g_ orbital is the SOMO in the ground state. The electrons in the orbitals of symmetry Ag are arranged in a singlet, and then they introduce a null spin density. The net spin density comes from the orbital 3a_u_, essentially localized on the π S_m_ orbitals and the C=C bond. Notice the similarity between the spin density plot for the 3^2^Au state (Figure 5) and the shape of the 3a_u_ orbital (Figure 4). This explains why the spin density on Cu atom in this state is dramatically reduced with respect to the ground state. Figure 5 shows the changes observed in the Mulliken spin population for atoms or groups of atoms with respect to the ground state. A negative (positive) +Δδ value means a reduction (increase) in the spin density on the center(s) with respect to the ground state. In the 3^2^Au state, a significant increase of the spin density on S_m_ sulfur atoms and C=C is observed together with a drastic reduction of the spin density on Cu atom.

A similar analysis can be done for the 6^2^Au state. In this case, the net spin density in the dominant configuration comes from the orbital 1a_u_, with a non-negligible contribution on the S_m_ sulfur atoms. This simple analysis is in agreement with the spin density plot of Figure 5, and the changes observed in the Mulliken spin population of the different groups of the system.

In the case of the state 7^2^Au, the spin density on Cu is 0.4231. Following the same approach, the net spin density on the 2a_g_ orbital can be roughly evaluated from the sum of the weights of the two former determinants of the dominant configuration (all having a spin up on the 2a_g_ orbital), minus the weight of the latter, where the 2a_g_ orbital bears a spin down. This weighted sum gives a spin density of 0.568 on the 2a_g_ orbital. Taking into account that this MO corresponds to a 65:35 mixing of Cu:dmit orbitals, the contribution to the Cu spin density is 0.369, to be compared with the Mulliken spin population value of 0.4231 (Table S3). The 3a_g_ and 1a_u_ orbitals carry the rest of the spin density for this dominant contribution; the corresponding spin density plot (Figure 5) presents similarities with the shape of these three open shell orbitals (2a_g_, 3a_g_, and 1a_u_).

For the rest of states where the analysis is much more complex, it is useful to look at the plots of the spin density change shown in Figure 5. The excited states 2^2^Au, 11^2^Au, and 12^2^Au present almost any change on the spin populations with respect to the ground state. In the rest of the explored states, a noticeable reduction of the spin density on Cu atom is observed in favor to the dmit ligand atoms. In short, the system in these states has a large character of organic radical. The increase of the dmit contribution to the spin density in those states accessible by UV–Vis is in agreement with the reduction of the average g value in the EPR spectra under irradiation [24] with g ~ 2.00 as expected for an organic radical. Among these excited states, five of them present non-negligible oscillator strength, then they are accessible by UV–Vis irradiation, and their population could be then responsible for the photoinduced changes reported for this system.

### 2.3. Magnetic Interactions

**Figure 6:**
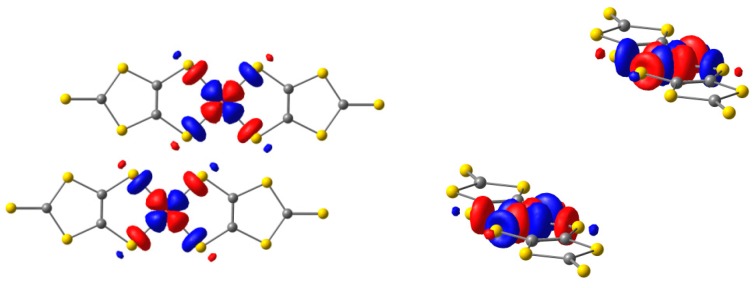
Top and side views of two neighbor [Cu(dmit)_2_]^−2^ complexes in the crystal, and the relative orientation of the SOMO on each complex. Yellow, red, and gray balls correspond to S, Cu, and C atoms, respectively.

The magnetic coupling between two neighbor [Cu(dmit)_2_]^−2^ complexes was evaluated by means of DDCI calculations. This approach is considered as the reference method in the field, providing estimates of the magnetic coupling in good agreement with the experimental data for most of the systems explored. Figure 6 shows how two close [Cu(dmit)_2_]^−2^ complexes are orientated in the crystal, occupying parallel planes, separated by a Cu…Cu distance of 8.466 Å. The shortest intermolecular S…S contact is as large as 6.14 Å.

The system contains two unpaired electrons in two orbitals (the in-phase and out-of-phase combinations of the SOMOs). The energy difference between the singlet and triplet states equals the magnetic coupling constant *J*, according to the previous definition of the Heisenberg Hamiltonian. At the DDCI level, the singlet and triplet states are almost degenerate states, the *J* value is just +0.12 cm^−1^ at this level imposing a precision of 10^−8^ Hartree to the energy. This very weak interaction is in agreement with both the long intermolecular distance and also the relative orientation of the SOMO orbitals on neighbor molecules, both preventing an efficient coupling between close units.

However the interaction in this system has been described as strongly antiferromagnetic and considered responsible for the diamagnetism observed for T >50 K. Naito and coworkers [23,24] relate the increase in χ at low temperature (T < 50–60 K) and the jumps in χ at ~50 K to the presence of impurities, such as oxygen adsorbed on the sample.

**Figure 7:**
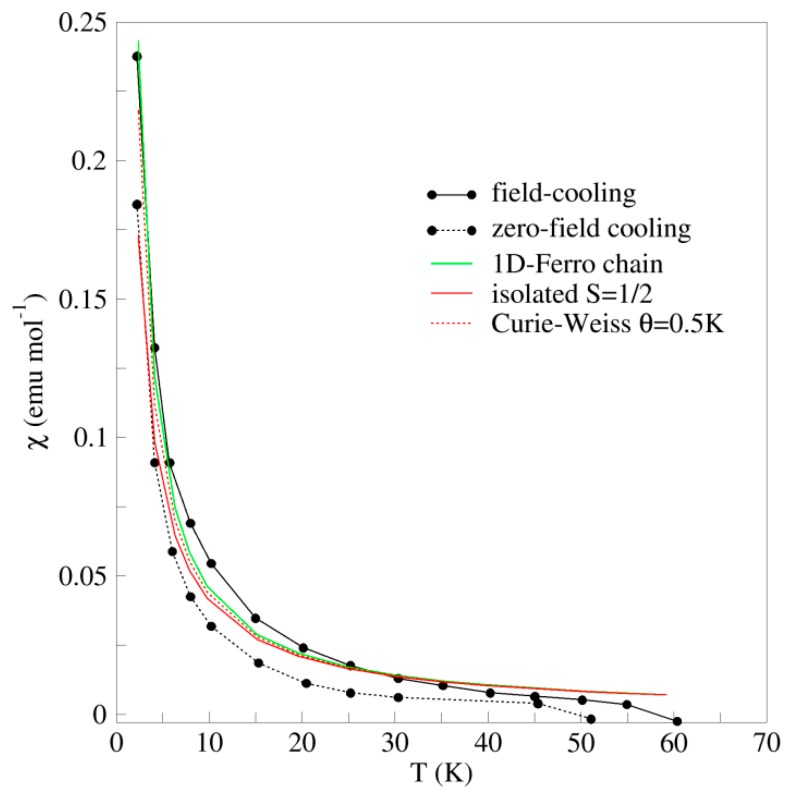
Temperature dependence of the magnetic susceptibility of [n-Bu_4_N]_2_[Cu(dmit)_2_] at low temperature. The experimental data (field-cooling and zero-field cooling) are adapted from Ref. [23]. Green curve corresponds to the 1D-ferromagnetic S = 1/2 chain [48], with g = 2.09 and J = 0.7 cm^−1^. Red curves represent the Curie law for the isolated S = 1/2 magnetic units (solid line) and the Curie–Weiss law with ferromagnetic Weiss constant θ = 0.5 K (dotted red line) (g = 2.09).

Our results indicate that these data can be interpreted in a different way. The presence of intermolecular interactions manifests in χ at low temperature, in particular, when these interactions are weak as in the present case. The temperature dependence of χ can be reproduced by assuming two different spin models. Since the coupling is very weak, it is possible to consider the [Cu(dmit)_2_]^−2^ as magnetically isolated S = 1/2 units, and fit the χ vs. T curve at low temperature using the Curie law, χ = N_A_g^2^β^2^S(S + 1)/3kT, with g = 2.09. This fitting corresponds to the red line in Figure 7. The weak ferromagnetic interaction between neighbor anions can be introduced by the mean field approximation, resulting in the Curie–Weiss law: χ = N_A_g^2^β^2^S(S + 1)/3k(T − θ), θ = zJS(S + 1)/3k. The dotted red line in Figure 7 corresponds to θ = 0.5 K (J = 0.7 cm^−1^ and g = 2.09). Alternatively, since the [Cu(dmit)_2_]^−2^ anions adopt a parallel arrangement along the *a* axis, it is possible to consider that they form isolated 1D-chains of ferromagnetically interacting [Cu(dmit)_2_]^−2^ complexes. The experimental χ data at low temperature can be fitted by using the expression by Baker et al. [48] for 1D ferromagnetic S = 1/2 chains:

$$\begin{array}{l} \chi =\frac{N_{A}g^{2}\beta^{2}}{4kT}{\left(\frac{A}{B}\right)}^{2/3}\\ A=1.0+5.7980x+16.9027x^{2}+29.3769x^{3}+29.8329x^{4}+14.0369x^{5}\\ B=1.0+2.7980x+7.0087x^{2}+8.6538x^{3}+4.5743x^{4}\\ x=J/2kT \end{array}$$

The resulting fitting χ vs. T curve is shown in Figure 7 (green line) for J = 0.7 cm^−1^ and g = 2.09. Notice that all fitted curves are in between the two reported sets of data for this compound registered following a zero-field cooling (dotted black line) and field-cooling processes (solid black line).

## 3. Discussion and Conclusions

The optical doping has been employed as alternative to the chemical oxidation for introducing unpaired electrons on the organic ligands in different salts of [Cu(dmit)_2_]^−2^ complex. This unit is a building block for many molecular magnetic and conductor systems, and the possibility of a direct control of the spin distribution by UV–Vis irradiation could be a remarkable step forward in the design of new molecular materials.

Our findings reveal the presence of several excited states where the spin density is (mainly) delocalized on the ligands, in contrast to the ground state where the spin density is largely localized on the Cu 3d orbital. Some of these states are accessible by an electronic excitation promoted by UV–Vis radiation, and then it is possible to optically control the spin distribution.

Our study based on CASSCF wave functions puts in evidence the complexity of the electronic structure of the low-lying states of [Cu(dmit)_2_]^−2^ complex and the versatility of the noninnocent dithiolene ligand. The key findings can be summarized as follows.

- The ground state SOMO shows strong dmit-Cu hybridization that is in agreement with previous characterizations [23,27,37,38]. The large Cu character of this orbital is in good agreement with the g-values resulting from the analysis of the EPR data in the dark.
- The excited states in the range of 2.0 to 5.5 eV present a noticeable multideterminantal character; the dominant configuration represents in most of the states no more than 50% of the whole wave function. This is a consequence of the presence of numerous virtual dmit π orbitals very close in energy, in such a way that the population of these empty orbitals by excitations from the occupied orbitals requires almost the same input of energy. As a result, in most of the explored states the spin density moves from the Cu 3d_xy_ to the dmit π orbitals. This multideterminantal character makes difficult a proper description by means of single-reference methods such as DFT, TD-DFT and, of course, extended Hückel calculations, and could be in the origin of the discrepancies found between our analysis and those previously reported.
- Among these states, five of them present non-negligible oscillator strength, and then the excitations from the ground state are allowed by the electric-dipole selection rules. In these states, the spin density shows a marked change with respect to the ground state, i.e., it is mainly (or completely) localized on the dmit ligands. This feature agrees with the fact that the observed g-values (~2.00) under UV irradiation are comparable with that of the free electron, then in better agreement with the unpaired electrons placed on the organic dmit ligands than localized on the Cu centers.
- The magnetic interactions between the [Cu(dmit)_2_]^−2^ complexes are very weak, slightly ferromagnetic, and in good agreement with both the long intermolecular distances, and the relative orientation of the SOMO orbitals in two close [Cu(dmit)_2_]^−2^ complexes. The experimental thermal dependence of the magnetic susceptibility data at low temperature (T < 50 K) can be simulated assuming isolated S = 1/2 units interacting with a very weak ferromagnetic coupling, by a Curie–Weiss law or a 1D-ferromagnetic chain spin model. The reported diamagnetic behavior found for T > 50 K for these salts is by no means due to a strong antiferromagnetic interaction between the [Cu(dmit)_2_]^−2^ units, as claimed in previous works [23,24]. Neither the J coupling value nor the crystal structure supports such a strong interaction.

Since the UV–Vis spectra of the three explored [Cu(dmit)_2_]^−2^ salts and the changes found in the EPR spectra under irradiation are markedly similar, the conclusions of this work devoted to the [n-Bu_4_N]_2_[Cu(dmit)_2_] should be also relevant for other related salts of [Cu(dmit)_2_] showing photoinduced changes of the spin distribution. This study highlights the key role of accurate and careful theoretical studies based on state-of-the-art approaches to help better understand the properties of these complex systems and the changes induced by external stimuli.

## 4. Materials and Methods

All electron single point calculations were carried out on the [Cu(dmit)_2_]^−2^ complex, using the CASSCF [49] and CASPT2 [50,51] approaches, in order to obtain the electronic structure and energy differences of the ground and excited states. The geometries of the [Cu(dmit)_2_]^−2^ monomer and dimer complexes were directly obtained from the resolved X-ray structure of the crystal at 100 K under dark conditions [22]. It is worth mentioning that no geometrical modification has been observed in the irradiated structure [22]. The X-ray geometry of the [Cu(dmit)_2_]^−2^ complex presents an inversion centre (C_i_ point group), retained in all the calculations.

The active spaces used to describe the ground and excited states of the [Cu(dmit)_2_]^−2^ complex are the same in nature, although optimized independently. The CAS contains nine electrons in nine MOs: four belonging to the irreducible representation a_g_ and five to the a_u_ (Figure 4). The composition of the CAS has been chosen to include the low-lying excited stated accessible by UV–Vis irradiation. They correspond essentially to π and π * orbitals centered on the dmit ligands, together with the SOMO and the σ_1_+ σ_1_ dmit orbital. The excitations involving Cu 3d-like orbitals (d → SOMO, d → d) are out of the energy range accessible by UV–Vis radiation (mostly in the red-NIR range, see Table S3) and correspond to electric-dipole-forbidden transitions. For these reasons, they are not included in the determination. Only those excitations allowed by the electric transition dipole rules are computed, which in fact correspond to excitations between the ground ^2^Ag state and the excited ^2^Au states.

The oscillator strengths are determined using the Restricted Active Space State Interaction (RASSI) approach[52] on the basis of the CASPT2 description of the ground state and multi-state(MS)-CASPT2 wave functions for the Au states [51]. These wave functions are based on state-averaged CASSCF(9/9) MOs of the fifteen lowest doublet states of symmetry a_g_ and a_u_, respectively.

Finally, the magnetic coupling constant between two neighbor [Cu(dmit)_2_]^−2^ complexes has been also evaluated by means of Difference Dedicated Configuration Interaction (DDCI) [53,54] calculations on the singlet and triplet states, resulting from combining two unpaired electrons in two orbitals. This approach, considered as the reference method to evaluate magnetic coupling constants [55], takes into account all the excitations contributing to the energy difference between the magnetic states. It is possible to demonstrate, using second-order perturbation arguments, that the energy differences are correctly evaluated with the CI space restricted to single and double excitations involving at least one active orbital (i.e., inactive double excitations are excluded). The magnetic coupling constant was evaluated as the difference energy between the singlet and triplet states, J = E(S) − E(T), assuming that the interaction between the two unpaired electrons can be described by an isotropic Heisenberg Hamiltonian, $H=-\sum^{\;}J_{\mathrm{ij}}S_{i}S_{j}$ where S_i_, S_j_ corresponds to the spin operators of the two interacting sites i and j, respectively, and J_ij_ is the magnetic coupling constant between these sites. The procedure requires a common set of molecular orbitals. Here, the triplet CASSCF (4/6) MOs are employed in the DDCI calculations. The active space of this CASSCF calculation contains both the SOMOs and the corresponding in-phase combinations of the σ_1_ dmit orbitals and the Cu 3d_xy_ orbital, but only the SOMOs are considered as active space in the DDCI calculations.

Atomic Natural Orbital with Relativistic Core Correction (ANO-RCC) [56,57] basis set of polarized triple-zeta quality has been used with the following contractions; [5s4p3d1f] for Cu, [5s4p2d] for S, and [4s3p1d] for C atoms. CASSCF/CASPT2/RASSI calculations were performed with the MOLCAS@UU package [58], while for DDCI calculations we used the CASDI code by Maynau and coworkers [59,60].

## Figures and Tables

**Table 1:** Lowest excited Au states accessible by UV irradiation. Relative MS-CASPT2 energy (eV), wavelength (nm), oscillator strength (*f*), dominant component of the wave function, and Mulliken spin density on Cu atom (δ_Cu_) evaluated from the natural MOs for each root.

	ΔE	λ	*f*	Dominant Component of the Wave Function *	Weight (%)	δ_Cu_
X ^2^Ag	0			|2a00 22200|	84	0.8909
1 ^2^Au	1.97	628.2	0.83 × 10^−4^	------------------		−0.2843
2 ^2^Au	2.25	**551.2**	**0.738**	$-\sqrt{\frac{2}{3}}\left|aa00\;222b0\right|+\frac{1}{\sqrt{6}}\left[\left|ab00\;222a0\right|+\left|ba00\;222a0\right|\right]$	54.4	0.8830
3 ^2^Au	3.30	**375.7**	**0.026**	$\frac{1}{\sqrt{2}}\left[\left|2ab0\;22a00\right|-\left|2ba0\;22a00\right|\right]$	59.8	0.0501
4 ^2^Au	3.46	**358.7**	**0.014**	$-\sqrt{\frac{2}{3}}\left|2aa0\;22b00\right|+\frac{1}{\sqrt{6}}\left[\left|2ab0\;22a00\right|+\left|2ba0\;22a00\right|\right]$	58.6	0.4909
5 ^2^Au	3.52	352.3	0.24 × 10^−4^	------------------		−0.2852
6 ^2^Au	4.05	306.5	0.48 × 10^−2^	$\frac{1}{\sqrt{2}}\left[\left|2ab0\;a2200\right|-\left|2ba0\;a2200\right|\right]$	87.9	−0.0730
7 ^2^Au	4.07	**304.7**	**0.023**	$-\sqrt{\frac{2}{3}}\left|2aa0\;b2200\right|+\frac{1}{\sqrt{6}}\left[\left|2ab0\;a2200\right|+\left|2ba0\;a2200\right|\right]$	85.2	0.4231
8 ^2^Au	4.50	275.7	0.15 × 10^−5^	------------------		−0.2821
9 ^2^Au	4.66	266.1	0.47 × 10^−2^	$\frac{1}{\sqrt{2}}\left[\left|2ab0\;2a200\right|-\left|2ba0\;2a200\right|\right]$	58.2	0.6138
10 ^2^Au	4.73	**262.08**	**0.015**	$-\sqrt{\frac{2}{3}}\left|2aa0\;2b200\right|+\frac{1}{\sqrt{6}}\left[\left|2ab0\;2a200\right|+\left|2ba0\;2a200\right|\right]$	55.6	0.0566
11 ^2^Au	4.80	**258.6**	**0.564**	$-\sqrt{\frac{2}{3}}\left|aa00\;2220b\right|+\frac{1}{\sqrt{6}}\left[\left|ab00\;2220a\right|+\left|ba00\;2220a\right|\right]$	18.0 **	0.8357
12 ^2^Au	5.36	231.4	0.28 × 10^−2^	$-\sqrt{\frac{2}{3}}\left|0aa0\;2220b\right|+\frac{1}{\sqrt{6}}\left[\left|0ab0\;2220a\right|+\left|0ba0\;2220a\right|\right]$ $-\sqrt{\frac{2}{3}}\left|0a0a\;222b0\right|+\frac{1}{\sqrt{6}}\left[\left|0a0b\;222a0\right|+\left|0b0a\;222a0\right|\right]$	19.1 ** 16.9 **	0.8023
13 ^2^Au	5.44	227.7	0.72 × 10^−4^	------------------		−0.1959
14 ^2^Au	5.55	223.6	0.12 × 10^−3^	$\frac{1}{\sqrt{2}}\left[\left|ab20\;22a00\right|-\left|ba20\;22a00\right|\right]$ $\frac{1}{\sqrt{2}}\left[\left|ab00\;22a20\right|-\left|ba00\;22a20\right|\right]$	23.1 23.3	0.0431
15 ^2^Au	5.89	210.6	0.64 × 10^−5^	------------------		−0.2852

* The electronic configurations are expressed on the basis of the active MOs: (π_cc_ − π_cc_) (3d_xy_ + L) (π_cs_ − π_cs_) (π_cc_* + π_cc_*) (σ_1_ + σ_1_) (π_Sm_ − π_Sm_) (π_cc_ + π_cc_) (π_CS*_ + π_CS*_)(π_CC_* − π_CC_*) (the first four orbitals are of symmetry Ag, the rest are of symmetry Au). Only the dominant component of the states with *f* larger than 0.0001 is reported. ** Strongly multiconfigurational state, many contributions with small weight (5%).

## Supplementary Materials

The following are available online, Table S1: Lowest excited Au states accessible by UV irradiation. Relative MS-CASPT2 energy (eV), wavelength (nm), oscillator strength (*f*), dominant components of the wave function, and Mulliken spin density on Cu atom (δ_Cu_) evaluated from the natural MOs for each root, Table S2: Total spin density (Mulliken spin population analysis) on the Ag ground state (state #0) and Au excited states resulting from the CASPT2 and MS-CASPT2(9/9) calculations, respectively, Table S3. Relative energy and associated wavelength of the d → d states at SA-CASSCF (13e/7MO) (average of the five 3d states) and MS-CASPT2 levels. Table S4. Relative MS-CASPT2 (17e/14MO) energy (eV), wavelength (nm), oscillator strength (*f*), and dominant component of the wave function, evaluated from the natural MOs for each root. Figure S1. Active orbitals employed in the test CASSCF/CASPT2 (17e/14MO) calculations of the ground and excited doublet states of the [Cu(dmit)_2_]^−2^ complex.
