# Supplementary material for: Light-Induced Control of the Spin Distribution on Cu–Dithiolene Complexes: A Correlated Ab Initio Study
Source: Molecules. 2019 Mar 19;24(6):1088. doi: 10.3390/molecules24061088 (PMC6470791; doi:10.3390/molecules24061088)

Supporting Information

Light-Induced Control of the Spin Distribution on Cu–Dithiolene Complexes: A Correlated Ab Initio Study

Jhon Zapata-Rivera [1] and Carmen J. Calzado [2,*]

[1] Facultad de Ciencias Básicas and Universidad Tecnológica de Bolívar, Campus Tecnológico s/n, 131001 Cartagena, Colombia; jzapatarivera@icloud.com

[2] Departamento de Química Física, Universidad de Sevilla, c/ Prof. García González and s/n, 41012 Sevilla, Spain

* Correspondence: calzado@us.es

Table S1. Lowest excited A_u states accessible by UV-irradiation. Relative MS-CASPT2 energy (eV), wavelength (nm), oscillator strength (*f*), dominant components of the wavefunction, and Mulliken spin density on Cu atom (δ_{Cu}) evaluated from the natural MOs for each root.

Table S2. Total spin density (Mulliken spin population analysis) on the Ag ground state (state #0) and Au excited states resulting from the CASPT2 and MS- CASPT2(9/9) calculations, respectively.

Table S3. Relative energy and associated wavelength of the d→ d states at SA-CASSCF(13e/7MO) (average of the five 3d states) and MS-CASPT2 levels.

Table S4. Relative MS-CASPT2(17e/14MO) energy (eV), wavelength (nm), oscillator strength (*f*), and dominant component of the wavefunction, evaluated from the natural MOs for each root

Figure S1. Active orbitals employed in the test CASSCF/CASPT2(17e/14MO) calculations of the ground and excited doublet states of the $[Cu(dmit)_2]^{-2}$ complex.

Table S1. Lowest excited A_u states accessible by UV-irradiation. Relative MS-CASPT2 energy (eV), wavelength (nm), oscillator strength (*f*), dominant component of the wavefunction, and Mulliken spin density on Cu atom (δ_{Cu}) evaluated from the natural MOs for each root.

	ΔE	λ	*f*	Dominant component of the wavefunction*	Weight (%)	δ_{Cu}
X ^2A_g	0			$\vert 2a00\ 22200 \vert$	84	0.8909
1 ^2A_u	1.97	628.2	0.83E-4			-0.2843
2 ^2A_u	2.25	**551.2**	**0.738**	$-\sqrt{\frac{2}{3}}\vert aa00\ 222b0\vert + \frac{1}{\sqrt{6}}\left[\vert ab00\ 222a0\vert + \vert ba00\ 222a0\vert\right]$	54.4	0.8830
				$-\sqrt{\frac{2}{3}}\vert 2aa0\ 22b00\vert + \frac{1}{\sqrt{6}}\left[\vert 2ab0\ 22a00\vert + \vert 2ba0\ 22a00\vert\right]$	17.4	
3 ^2A_u	3.30	**375.7**	**0.026**	$\frac{1}{\sqrt{2}}\left[\vert 2ab0\ 22a00\vert - \vert 2ba0\ 22a00\vert\right]$	59.8	0.0501
				$-\sqrt{\frac{2}{3}}\vert aa00\ 222b0\vert + \frac{1}{\sqrt{6}}\left[\vert ab00\ 222a0\vert + \vert ba00\ 222a0\vert\right]$	20.0	
4 ^2A_u	3.46	**358.7**	**0.014**	$-\sqrt{\frac{2}{3}}\vert 2aa0\ 22b00\vert + \frac{1}{\sqrt{6}}\left[\vert 2ab0\ 22a00\vert + \vert 2ba0\ 22a00\vert\right]$	58.6	0.4909
				$\frac{1}{\sqrt{2}}\left[\vert ab00\ 222a0\vert - \vert ba00\ 222a0\vert\right]$	24.6	
5 ^2A_u	3.52	352.3	0.24E-4			-0.2852
6 ^2A_u	4.05	306.5	0.48E-2	$\frac{1}{\sqrt{2}}\left[\vert 2ab0\ a2200\vert - \vert 2ba0\ a2200\vert\right]$	87.9	-0.0730
7 ^2A_u	4.07	**304.7**	**0.023**	$-\sqrt{\frac{2}{3}}\vert 2aa0\ b2200\vert + \frac{1}{\sqrt{6}}\left[\vert 2ab0\ a2200\vert + \vert 2ba0\ a2200\vert\right]$	85.2	0.4231
8 ^2A_u	4.50	275.7	0.15E-5			-0.2821
9 ^2A_u	4.66	266.1	0.47E-2	$\frac{1}{\sqrt{2}}\left[\vert 2ab0\ 2a200\vert - \vert 2ba0\ 2a200\vert\right]$	58.2	0.6138
				$-\sqrt{\frac{2}{3}}\vert 2aa0\ 2b200\vert + \frac{1}{\sqrt{6}}\left[\vert 2ab0\ 2a200\vert + \vert 2ba0\ 2a200\vert\right]$	33.4	
10 ^2A_u	4.73	**262.08**	**0.015**	$-\sqrt{\frac{2}{3}}\vert 2aa0\ 2b200\vert + \frac{1}{\sqrt{6}}\left[\vert 2ab0\ 2a200\vert + \vert 2ba0\ 2a200\vert\right]$	55.6	0.0566
				$\vert\ \frac{1}{\sqrt{2}}\left[\vert 2ab0\ 2a200\vert - \vert 2ba0\ 2a200\vert\right]$	33.8	
11 ^2A_u	4.80	**258.6**	**0.564**	$-\sqrt{\frac{2}{3}}\vert aa00\ 2220b\vert + \frac{1}{\sqrt{6}}\left[\vert ab00\ 2220a\vert + \vert ba00\ 2220a\vert\right]$	18.0**	0.8357
12 ^2A_u	5.36	231.4	0.28E-2	$-\sqrt{\frac{2}{3}}\vert 0aa0\ 2220b\vert + \frac{1}{\sqrt{6}}\left[\vert 0ab0\ 2220a\vert + \vert 0ba0\ 2220a\vert\right]$	19.1**	0.8023
				$-\sqrt{\frac{2}{3}}\vert 0a0a\ 222b0\vert + \frac{1}{\sqrt{6}}\left[\vert 0a0b\ 222a0\vert + \vert 0b0a\ 222a0\vert\right]$	16.9**	
13 ^2A_u	5.44	227.7	0.72E-4			-0.1959
14 ^2A_u	5.55	223.6	0.12E-3	$\frac{1}{\sqrt{2}}\left[\vert ab20\ 22a00\vert - \vert ba20\ 22a00\vert\right]$	23.1	0.0431
				$\frac{1}{\sqrt{2}}\left[\vert ab00\ 22a20\vert - \vert ba00\ 22a20\vert\right]$	23.3	
15 ^2A_u	5.89	210.6	0.64E-5			-0.2852

*The electronic configurations are expressed on the basis of the active MOs, ordered as follows: **(π_{cc}-π_{cc})($3d_{xy}$+L)**(π_{cs}-π_{cs})**(π_{cc}*+π_{cc}*)** (σ_1+σ_1) (π_{Sm}-π_{Sm}) (π_{cc}+π_{cc}) (π_{CS^*}+π_{CS^*})(π_{CC}*-π_{CC}*) (in bold the symmetry A_g MOs, the rest are of symmetry A_u). Only the dominant component of the states with *f* larger than 0.0001 is reported. ** Strongly multiconfigurational state, many contributions with small weight (5%)

Table S2. Total spin density (Mulliken spin population analysis) on the Ag ground state (state #0) and Au excited states resulting from the CASPT2 and MS- CASPT2(9/9) calculations, respectively.

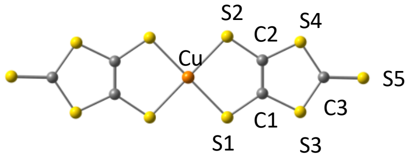

State	Cu	S1	S2	S3	S4	S5	C1	C2	C3
0	0,8909	0,0256	0,0262	0,0001	0,0001	-0,0001	0,0013	0,0013	0,0000
1	-0,2843	0,0549	0,0457	0,0555	0,0580	0,0932	0,0855	0,0886	0,1608
2	0,8831	0,0243	0,0246	0,0014	0,0014	0,0025	-0,0007	-0,0007	0,0056
3	0,0501	0,1220	0,0647	0,0223	0,0289	0,0334	0,0676	0,0905	0,0457
4	0,4908	-0,0102	-0,0035	0,0346	0,0356	0,0725	-0,0092	-0,0079	0,1426
5	-0,2852	0,0719	0,0637	0,0288	0,0309	0,0240	0,2007	0,2020	0,0207
6	-0,0730	0,1302	0,1011	0,0319	0,0308	0,0399	0,0735	0,0672	0,0618
7	0,4231	-0,0080	-0,0020	0,0361	0,0372	0,0753	0,0013	0,0032	0,1450
8	-0,2821	0,0417	0,0334	0,0627	0,0652	0,1039	0,0717	0,0747	0,1877
9	0,6138	0,2716	0,0384	-0,0124	-0,0163	-0,0305	0,0105	0,0025	-0,0708
10	0,0566	0,0028	0,0028	0,0586	0,0585	0,1169	0,0039	0,0023	0,2259
11	0,8357	0,0259	0,0252	0,0035	0,0035	0,0060	0,0034	0,0031	0,0115
12	0,8023	0,0323	0,0315	-0,0014	-0,0013	-0,0081	0,0323	0,0321	-0,0184
13	-0,1959	0,0486	0,0419	0,0479	0,0501	0,0707	0,1080	0,1098	0,1210
14	0,0431	0,0835	0,0938	0,0363	0,0275	0,0451	0,0739	0,0438	0,0745
15	-0,2851	0,0743	0,0637	0,0278	0,0303	0,0199	0,2061	0,2084	0,0120

Table S3. Relative energy and associated wavelength of the d→ d states at SA-CASSCF(13e/7MO) (average of the five 3d states) and MS-CASPT2 levels.

E(CASSCF)/eV	λ / nm	E(MS-CASPT2)/eV	λ /nm
0		0	
1.16	1069	1.76	704
1.23	1008	1.85	670
1.24	1000	1.95	636
1.30	954	1.99	623

Table S4. Relative MS-CASPT2(17e/14MO) energy (eV), wavelength (nm), oscillator strength (*f*), and dominant component of the wavefunction, evaluated from the natural MOs for each root. Remaining doublet states have been not included because they have negligible oscillator strength. The determinants are written following the order $|3d_{xz}3d_{xy}3d_{x2-y2}3d_{z2}3d_{yz}(\pi_{CC}-\pi_{CC})(\pi_{CC}+\pi_{CC})\ (\pi_{Sm}-\pi_{Sm})(\sigma_1-\sigma_1)\ 4d_{x2-y2}4d_{xy}4d_{z2}4d_{yz}\ 4d_{xz}|$ and are represented in Figure S1.

	ΔE	λ	*f*	Dominant component of the wavefunction*
X ^{2}A	0			63%|2a222222200000|
2 ^{2}A	2.28	544.6	0.0006	95%|222222a2200000|
7 ^{2}A	3.24	382.9	0.1833	95%|2222222a200000|
8 ^{2}A	4.51	275.0	0.0000	63%|22222222a00000|

Figure S1. Active orbitals employed in the test CASSCF/CASPT2(17e/14MO) calculations of the ground and excited doublet states of the $[Cu(dmit)_2]^{-2}$ complex. The occupation represents the dominant configuration of the ground state. The calculations have been performed without symmetry for this reason the orbital ($\pi_{Sm}-\pi_{Sm}$) is slightly distorted.

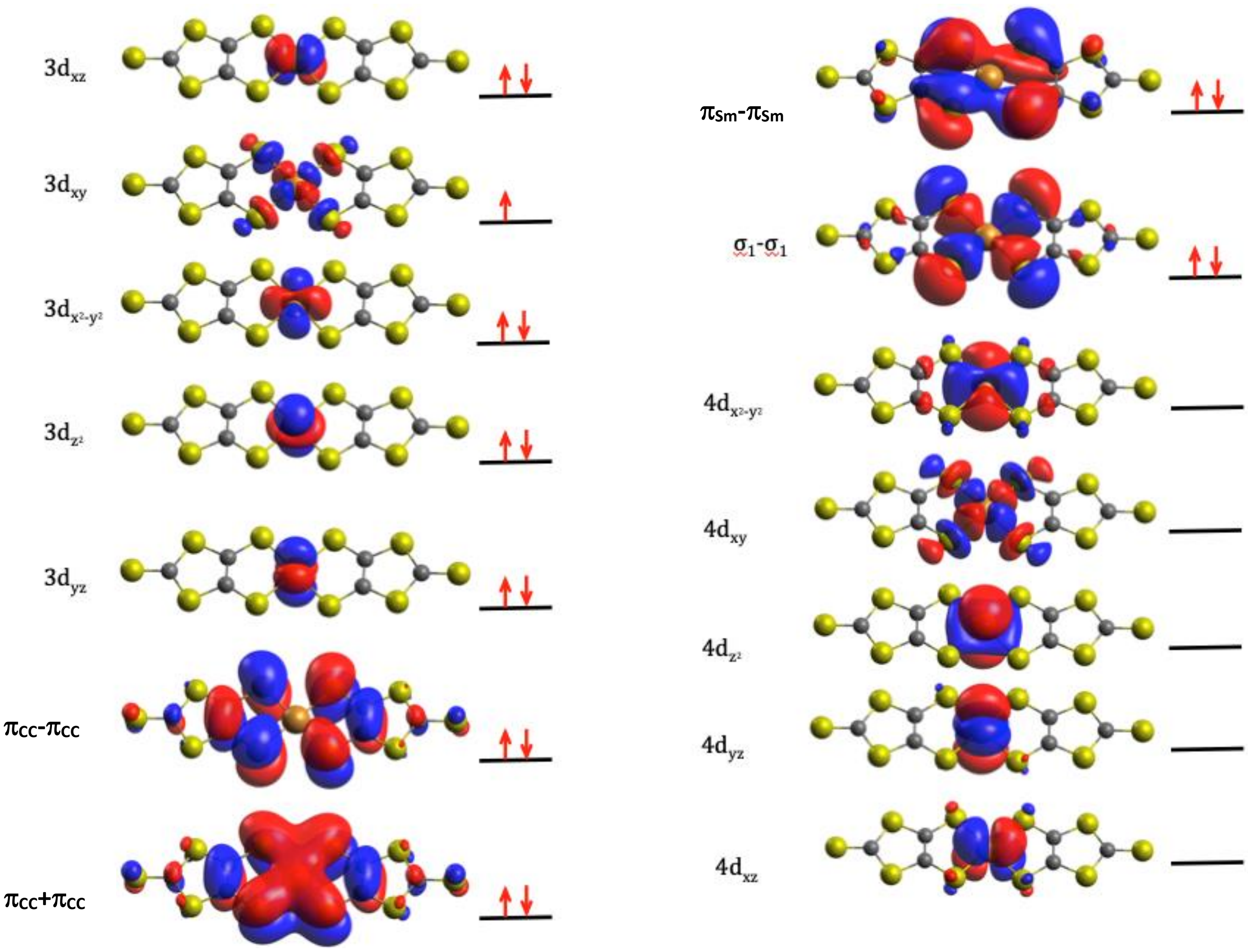

Supplement: Supplementary file 1 [file molecules-24-01088-s001.pdf]
